# Health Diplomacy and Ethical Considerations for Deployed Military Clinicians Managing Tensions Between Local National Groups

**DOI:** 10.1111/dewb.70040

**Published:** 2026-05-21

**Authors:** Janet Kelly, Tom Falconer‐Hall, John Whitaker

**Affiliations:** ^1^ School of Nursing and Midwifery, Faculty of Health and Social Care University of Hull HU6 7RX Hull UK; ^2^ DMS Centre for Defence Engagement Research and Clinical Innovation Birmingham UK; ^3^ Institute of Applied Health Science University of Birmingham Birmingham UK; ^4^ Academic Department of Military Surgery and Trauma Royal Centre for Defence Medicine Birmingham UK

**Keywords:** diplomacy, harm, health care, impartiality, negotiation, obligation, politics, public relations

## Abstract

Military clinicians in disaster relief must treat health diplomacy not just as an operational tool but as a potential ethical obligation that actively prevents the political misuse of healthcare in situations of acute ethical tension. In settings where host nation military actors may threaten vulnerable populations, clinicians assume dual role**s** as caregivers but also as ethical diplomats often acting as an advocacy for at risk groups. This paper argues that military clinicians must treat health diplomacy as an active ethical responsibility rather than solely a neutral operational function. It suggests it is essential to prevent the political exploitation of healthcare and protect vulnerable groups in unstable and challenging environments. The paper uses an adapted four‐pillars framework to analyse challenges of impartiality, host‐nation influence and ethnic bias. It examines how clinicians negotiate complex health‐diplomacy environments, especially where vulnerable groups may face racially motivated harm.

## 1 Introduction

### 1.1 Defining Health Diplomacy in Military Humanitarian Engagements

Health diplomacy can include governments intervening to support public health and healthcare in partner countries whilst simultaneously advancing their own foreign policy goals [1]. The term strategic health diplomacy can describe situations where foreign policy goals such as security or economic interests take primacy, whereas ethical health diplomacy can describe situations where the motivation to achieve equitable and just access to healthcare predominates [2]. The term global health diplomacy acknowledges legitimate dual goals of improving both health and international relations concurrently [2]. Deployed military clinicians can therefore expect to function with dual roles as both healthcare providers and, as military personnel, diplomatic actors. In politically charged contexts, this duality introduces moral risks [3]. The clinician's responsibility must prioritise medical ethical principles over political considerations, especially when health diplomacy may be exploited to legitimise hostile actions against marginalised groups. Ethical decision‐making must resist being diluted by diplomatic expediency.

In this scenario the deployed clinician is faced with an environmental disaster (earthquake and tsunami) leading to displaced persons, compounded by the sequalae of multimodal infectious disease outbreaks. As well as concerns that some within the Host Nation (HN) military may wish to exploit the context to advance persecution of a HN ethnic minority.

This short essay seeks to discuss how the deployed clinician could frame the ethical issues to manage this situation.

### 1.2 Identifying Ethical Implications Using an Adapted “4 Pillars” Framework

The four pillars of biomedical ethics: beneficence, non‐maleficence, autonomy, and justice, developed by Beauchamp and Childress in 1979 [4], are familiar to most clinicians for approaching ethical considerations in individual patient management. In any global health engagement (GHE) scenario, the four pillars provide a potentially useful framework for organising and managing the key ethical issues. However, these principles require further interpretation in the complex ethical context of military humanitarian deployments [5]. When considering HN beneficence (doing good) this includes medical indications for interventions, expected benefits to the population and the likelihood of wider dissemination. Beneficence includes both improved health outcomes and broader societal benefits. Autonomy incorporates respecting community‐level agency and the rights of displaced or persecuted populations. HN Autonomy includes interventions paying heed to preferences and needs within the country, whilst being HN led and governed. Non‐maleficence demands scrutiny of how military logistics and power dynamics may enable or legitimise harm to vulnerable groups. Doing no harm to the HN considers risks and burdens placed on the local population, how sustainable an intervention might be, and whether impacts might be delayed or short lived. Justice compels clinicians to be proactive in identifying, challenging, and mitigating inequitable access to care, resisting propagation of health disparities. In this scenario, HN justice must consider the potential social, cultural, religious and economic effects and how they might impact equality and equity. This includes considering cost and wider societal social justice benefits. In military settings, these pillars are applied by the deployed clinician shaping ethical medical governance under pressure, no easy task.

In this scenario, the deployed clinician should consider whether increased HN military access to the camp would improve public health outcomes. HN actors are likely to better understand and influence local cultural practices, and their role within disease transmission, which might offer benefit in getting control of the outbreaks. Cultural insight into burial practices and attitudes proved valuable in the 2014 West African Ebola crisis response for example [6]. Additionally, the security situation within the camp appears to have deteriorated. Depending on existing camp security, additional military presence could deter further violence and its adverse health effects, as well as enforce any extant public health interventions including curfews or social distancing, and facilitating orderly vaccination programmes [7].

HN autonomy would include supporting the HN taking the leading role in governing the crisis response as well as other enduring pillars of government such as housing, sanitation, security and healthcare [8]. Furthermore, the foreign military forces are likely to be deployed following invitation from the HN. Therefore, they may lack legal authority, specifically under local domestic law, to prevent the HN military accessing the camp. The given context is one of considerable dependence of the HN on external actors, including the foreign militaries, but also other unspecified international organisations. If other HN governmental actors without similar concerns regarding ethnic minority bias exist, they might be approached to provide more HN camp governance and leadership [7]. Expressions of HN autonomy may also be nuanced by historical contexts not explicit in the scenario. Deployed clinicians should be mindful of the persistence or perception of imbalances in power structures that could shape HN actors' desire to cooperate or take guidance from a foreign military [9].

Non‐maleficence considerations here include risk of mistreatment of ethnic minorities being repeated. A HN government body's behaviour towards different populations may seem difficult for a deployed clinician to influence. Concerns could be raised within trusted bodies or persons within the HN, to understand whether the concerns are founded and what could be done to mitigate those concerns. Other stakeholders within the multinational multiagency response should be involved. Explicitly neutral non‐governmental organisations will likely have a perspective on the risk of harm and possible mitigations. Bodies such as the International Committee of the Red Cross could take a lead role in monitoring interactions on camp in the short to medium term [10]. Diplomatic actors could frame refusal to grant access as a compliance issue with established norms. If stakeholders could agree on the degree of involvement of the HN military in running the camp, risks could be mitigated through implementing alternative security measures such as joint patrols with other agencies that do not compromise minoritised populations. Whilst a deployed clinician may be limited in their ability to influence broad systemic change, failing to act or being complicit in unjust outcomes risks moral injury [11]. Moral injury can arise when clinicians are forced to act against their ethical beliefs, such as remaining silent in the face of persecution or facilitating access that enables abuse [12]. Ethical resilience requires clinicians to identify lines they will not cross and develop strategies to raise concerns diplomatically, through trusted intermediaries, or via coalition‐building with other agencies. HN complicity in discriminatory actions should not be normalised for the sake of maintaining diplomatic harmony. Silence can be ethically interpreted as tacit consent. Ethically courageous action whether refusing to share sensitive information, escalating to neutral oversight bodies, or documenting abuse for future accountability may be the only available protection for vulnerable groups.

Social justice concerns are front and centre of this ethical dilemma. The stress and hardship of a disaster response is likely to heighten pre‐existing tensions of any nature. Here ethnicity is the characteristic along which discrimination and maltreatment is feared. A deep understanding of the historical, religious, cultural, economic and other broader societal context is needed, to grasp the potential causes and risks of the situation [7, 13]. This scenario and its response offers the potential for broader impacts beyond the boundaries of the camp. As a microcosm of presumed wider inequalities based on ethnicity, an outcome of heightened persecution has potential to cause unrest and embed the inequalities further in future. Conversely, there is an opportunity for a well‐handled scenario to have a broader positive impact in addressing ethnic inequalities and wider national reconciliation. However, this too may risk moral injury in a deployed clinician, ultimately constrained in the influence they can exert, and who should not feel a burden of responsibility for the unjust actions of others [14]. Support channels, ethical escalation routes, and personal thresholds of acceptable conduct are helpful to mitigate the psychological burden for clinicians working in ethically compromised environments.

### 1.3 Countering Discrimination and Preventing Medical Stigmatisation

There are historical examples of infectious disease outbreaks and control measures being used to justify discrimination, so the concern within this scenario does have precedent in public health crises [15, 16]. Recent experience from the COVID 19 pandemic shows how narratives on disease can fuel persecution [17]. In today's information age of social media the importance of engaging with effective communication strategies is more important than ever. The principles are broadly the same for any military engagement and public health communication strategy. Engagement with local community leaders to understand and counter discriminatory narratives is key. Communication should include neutral, science‐based crisis response language to counter alternative discriminative misinformation [14]. Public health reporting should be transparent to ensure fair resource distribution [18]. In this scenario the deployed clinician has personal ethical responsibilities whilst carrying out their duties. They can set an example through upholding non‐discriminatory healthcare practices. The policies and practices enacted in the camp can explicitly require equitable treatment for all displaced persons, regardless of ethnicity. In this scenario a measles outbreak is established. A vaccination programme is a required intervention to help limit excess mortality and a carefully delivered communication strategy will be essential to counter vaccine hesitancy [7]. Military clinicians must actively counter discriminatory disease narratives, ensuring communication, vaccination, and surveillance strategies do not reinforce bias. This includes transparent data sharing, culturally competent messaging, and ensuring equitable resource access.

### 1.4 Navigating Cultural and Geopolitical Tensions in Humanitarian Crises

Understanding the context in any engagement operation is a prerequisite for maximal impact and minimising risk of causing harm. In this scenario the long term goals of foreign military engagement in the disaster relief operation are not expressed but should be understood by the deployed clinician [19]. Whilst a humanitarian objective to address human suffering is laudable, militaries are not independent humanitarian actors and the broader foreign policy agenda of the deployed clinician's military should be borne in mind [20]. To that end, were the scenario to deteriorate and ethnic discrimination and violence propagate, there is a real risk of the deployed clinician's military appearing complicit.

The same challenge exists at an individual patient management level, where both different medical treatment and human rights standards, may lead to divergent clinical care decisions [5]. Here, understanding strategic engagement goals can help influence the depth of engagement by the deployed clinical team. If the intention is for enduring and embedded partnerships, working towards pragmatic interoperability [21], additional training in ethical decision making could be delivered, with bespoke tools made available [22]. Similarly, as a negotiating tool, if the HN military wish to continue to rely on the deployed foreign militaries' assistance, requirements for strengthening medical governance structures to prevent political exploitation of health and care can be stipulated [5]. In pursuit of this goal and to avoid accusations of paternalism, accepted international standards, such as those set by the World Health Organisation, should be the target for strengthening [23]. These negotiations need not be in isolation, broader diplomatic engagement through other government departments can also mitigate discrimination. Multinational coalitions with military and civilian actors can together reinforce impartial medical care standards. Ethical engagement can include conditional cooperation with host militaries, advocacy for neutral oversight, and clear boundaries against coercion or unequal treatment.

Whilst we have chosen the four pillars as a framework to approach this scenario other frameworks exist. The humanitarian principles of humanity, neutrality, impartiality and independence are a useful cornerstone to guide humanitarian actors such as non‐governmental relief organisations. However, we take the view that it is inherently difficult for military actors, taking orders from their government, to truly act in a neutral, impartial or independent manner [20].

## 2 Conclusion: Health Diplomacy as an Ethical and Strategic Imperative

Within this complex and high‐risk scenario, we have discussed military clinicians as ethical and diplomatic actors. We have introduced a modification of the four pillars of biomedical ethics as a framework to consider pertinent ethical issues. Balancing medical ethics with operational and diplomatic imperatives poses challenges. Military engagement principles can shape ethical medical interventions in hostile environments. We recommend embedding engagement ethics training in pre‐deployment programmes, enhancing civil‐military cooperation with humanitarian agencies to strengthen ethical oversight, and integrating structured risk assessment tools into military engagement operations. Health diplomacy is not passive—it requires active negotiation, advocacy, and strategic engagement to prevent medical interventions from being exploited for potential abuse and discrimination.

## Conflicts of Interest

The authors declare no conflicts of interest.

## Data Availability

Data sharing not applicable to this article as no datasets were generated or analysed during the current study.
